# Knowledge and actions for the promotion and protection of breastfeeding by primary health care professionals in Uberlândia, 2022

**DOI:** 10.1590/S2237-96222026v35e20240245.en

**Published:** 2026-02-16

**Authors:** Amanda Borges Amaral, Vivian Mara Gonçalves de Oliveira Azeredo, Leandro Alves Pereira, Ana Elisa Madalena Rinaldi

**Affiliations:** 1 Universidade Federal de Uberlândia, Faculdade de Medicina, Uberlândia, MG, Brazil; 2 "> Universidade Federal de Uberlândia, Faculdade de Educação Física e Fisioterapia, Uberlândia, MG, Brazil; 3 "> Universidade Federal de Uberlândia, Instituto de Matemática e Estatística, Uberlândia, MG, Brazil

**Keywords:** Breast Feeding, Primary Health Care, Law Enforcement, Food and Nutritional Surveillance, Cross-Sectional Studies., Lactancia Materna, Atención Primaria de Salud, Aplicación de la Ley, Vigilancia Alimentaria y Nutricional, Estudios Transversales.

## Abstract

**Objective:**

To analyze, within the scope of primary health care, actions for the promotion and protection of breastfeeding, the knowledge and compliance with Law No. 11,265/2006, and the use of the National Food and Nutrition Surveillance System (*Sistema Nacional de Vigilância Alimentar e Nutricional*, SISVAN) by health professionals.

**Methods:**

Cross-sectional study with a probability sample of coordinators and health professionals. Two questionnaires were developed to collect data on professional training: knowledge and compliance with Law No. 11,265/2006; and knowledge and use of the Food and Nutrition Surveillance System. Data were expressed as relative frequency, and the chi-square test was used to compare them according to the model of care and professional training.

**Results:**

A total of 215 health professionals participated in the study, with only 12.4% reporting knowledge of Law No. 11,265/2006, and 30.4% describing its objectives. Conducting groups of pregnant women was the main action for promoting breastfeeding (82.3%), and most professionals (76.0%) managed it, focusing on latch and positioning. All coordinators and 77.7% of the professionals reported knowledge of the Food and Nutrition Surveillance System, while 86.2% of the coordinators and 45.7% of the professionals were able to describe its objective; only 27.6% of the coordinators used its data to develop actions in their unit.

**Conclusion:**

The majority of professionals are unaware of the main law protecting breastfeeding. Groups of pregnant women constitute the main action for promoting breastfeeding. Despite knowledge of the Food and Nutrition Surveillance System, its use as a basis for actions is insufficient.

## Resumen

**Objetivos:**

Analizar, en el ámbito de la atención primaria, las acciones de promoción y protección de la lactancia materna, el conocimiento y cumplimiento de la Ley N.º 11.265/2006 y el uso del Sistema Nacional de Vigilancia Alimentaria y Nutricional (*Sistema Nacional de Vigilância Alimentar e Nutricional*, SISVAN) por parte de los profesionales de la salud.

**Métodos:**

Estudio transversal, con muestra probabilística de coordinadores y profesionales de la salud. Se elaboraron dos cuestionarios para la recolección de datos sobre la formación profesional: conocimiento y cumplimiento de la Ley N.º 11.265/2006; y conocimiento y utilización del Sistema de Vigilancia Alimentaria y Nutricional. Los datos se expresaron en frecuencia relativa y se utilizó la prueba de chi-cuadrado para compararlos según el modelo de atención y la formación profesional.

**Resultados:**

Participaron del estudio 215 profesionales de la salud, de los cuales solo el 12,4% afirmó conocer la Ley N.º 11.265/2006, y el 30,4% describió sus objetivos. La realización de grupos de gestantes fue la principal acción de promoción de la lactancia materna (82,3%), y la mayoría de los profesionales (76,0%) realizaba su manejo, enfocándose en el agarre y la posición. Todos los coordinadores y el 77,7% de los profesionales afirmaron conocer el Sistema de Vigilancia Alimentaria y Nutricional, mientras que el 86,2% de los coordinadores y el 45,7% de los profesionales sabían describir su objetivo; solo el 27,6% de los coordinadores utilizaban sus datos para elaborar acciones en la unidad.

**Conclusión:**

La mayoría de los profesionales desconoce la principal ley de protección a la lactancia materna. Los grupos de gestantes constituyen la principal acción de promoción de la lactancia materna. A pesar del conocimiento sobre el Sistema de Vigilancia Alimentaria y Nutricional, su uso para fundamentar acciones es insuficiente.

**Ethical aspects**

This research respected ethical principles, having obtained the following approval data:

Research ethics committee: Universidade Federal de Uberlândia

Opinion number: 4,910,277

Approval date: 16/8/2021

Certificate of submission for ethical appraisal: 50201321.0.0000.5152

Informed consent record: Obtained from all participants prior to data collection.

## Introduction

In Brazil, since 2007, three strategies have been developed aimed at qualifying primary health care professionals for managing child nutrition ^1^ ^,^ ^2^ ^,^ ^3^. These strategies aim to improve work processes through critical-reflective education, with an emphasis on training tutors and conducting workshops. The Brazilian Breastfeeding Network (*Rede Amamenta Brasil*) aims to qualify health professionals for the promotion and management of breastfeeding ^2^. Additionally, in 2010, the National Strategy for Healthy Complementary Feeding (*Estratégia Nacional para Alimentação Complementar Saudável*, ENPACS) was proposed as a tool to support and strengthen actions focused on complementary feeding, especially in primary health care ^3^. In 2013, both were unified in the Brazilian Breastfeeding and Complementary Feeding Strategy (*Estratégia Amamenta e Alimenta Brasil*, EAAB) ^1^, aiming at the promotion and protection of breastfeeding and complementary feeding.

The certification of primary health care teams in the Brazilian Breastfeeding and Complementary Feeding Strategy (EAAB) is based on the monitoring of breastfeeding and complementary feeding indicators and compliance with the Brazilian Code of Marketing of Infant and Toddlers Food, Teats, Pacifiers and Baby Bottles (*Norma Brasileira de Comercialização de Alimentos para Lactentes e Crianças de Primeira Infância, Bicos, Chupetas e Mamadeiras*) ^1^. This standard was created from the International Code of Marketing of Breast-Milk Substitutes to regulate the marketing activities of infant food industries and related products ^4^. It was enacted as Law No. 11,265 in 2006 and regulated by Decree No. 9,579 in 2018 to protect breastfeeding ^5^ ^,^ ^6^. 

The monitoring of breastfeeding and complementary feeding indicators should be carried out from the Food and Nutrition Surveillance System (SISVAN), according to the guidelines of the Brazilian National Food and Nutrition Policy (*Política Nacional de Alimentação e Nutrição*) ^7^ and the National Policy for Integral Child Health Care (*Política Nacional de Atenção Integral à Saúde da Criança*) ^8^. Despite advances in recording dietary consumption markers between 2008 and 2019, the coverage and use of the system are still limited. Between 2008 and 2013 ^9^, coverage ranged from 0.1% to 0.4%, and between 2015 and 2019 ^10^, from 0.2% to 0.9%, with regional differences and higher coverage among children under four years old. 

The implementation of the Brazilian Breastfeeding and Complementary Feeding Strategy (EAAB) is not homogeneous among Brazilian municipalities ^11^ ^,^ ^12^. By 2019, approximately 48,000 health professionals ^13^ had participated in training; however, the strategy was consolidated in only 9.4% of the country’s health centers, with greater coverage in the Central-West and North regions ^14^.

Minas Gerais stood out for having the highest number of workshops conducted and tutors trained ^14^. In Uberlândia, Minas Gerais, only one workshop was held in 2017. In 2022, the city achieved the best performance in the Ministry of Health’s Final Composite Index among municipalities with over 500,000 inhabitants ^15^ ^,^ ^16^. Given this extensive coverage, it is necessary to analyze in detail the actions for the promotion and protection of breastfeeding in primary health care, the recommendations guiding professional practices, and professionals’ knowledge of essential components of the Brazilian Breastfeeding and Complementary Feeding Strategy (EAAB). Accordingly, this study aimed to analyze, within the scope of primary health care, the actions undertaken for the promotion and protection of breastfeeding, the knowledge and compliance with Law No. 11,265/2006, as well as the awareness and utilization of the Food and Nutrition Surveillance System by health care professionals.

## Methods

Study design and setting

This cross-sectional study was conducted in health centers and family health centers in Uberlândia, Minas Gerais, from January to July 2022. The population of the municipality is 713,222 inhabitants ^17^, and, in the year of data collection, primary health care coverage was 93.4% ^16^. In 2022, there were eight basic health units and 58 family health centers in the municipality, distributed in five health sectors (North/South/East/West/Central-West).

Participants

The study population included health professionals working in primary health care who assist children under two years old and/or pregnant women, comprising physicians, nurses, nutritionists, nursing technicians, as well as professionals serving as center coordinators at the time of the research.

Variables and data measurement

Two questionnaires were developed specifically for this study, organized into four sections, one aimed at health professionals and the other at center coordinators. The choice of open-ended questions is justified by the two central objectives of the study: knowledge of Law No. 11,265/2006 and the Food and Nutrition Surveillance System (SISVAN), and the implementation of breastfeeding promotion and protection actions carried out by health professionals. The open-ended questions helped us avoid influencing the response of the participants. The two questionnaires were tested in a pilot study conducted at a health center in 2021 to assess the application time and the conformity of professionals’ responses to the questions asked. For each question of the questionnaire, the expected response was described. When professionals’ responses did not align with the expected answers, the question was modified to obtain the desired content. The pilot study and data collection were conducted by a single researcher to standardize data collection and to gain familiarity with the physical structure of the centers. One of the questionnaire items included direct observation of possible advertisements for infant formulas and related products. During data entry and analysis, the consistency of the responses was examined. There were no missing answers in the questionnaires. The first section of the questionnaire included demographic data (age), educational background (profession and completion of *lato sensu* postgraduate studies [specialization, residency]) (yes/no), and professional activity for both center coordinators and professionals-length of service in the public health system (months), use of materials for breastfeeding updates (yes/no), participation in courses on breastfeeding management (yes/no-health professionals only), participation in service management courses (yes/no-center coordinators only), and participation in training workshops for the Brazilian Breastfeeding and Complementary Feeding Strategy (EAAB) (yes/no).

The second section included questions about knowledge of Law No. 11,265/2006 and its compliance in health centers (yes/no). For professionals who answered “yes,” an open-ended question asked whether they knew the objectives of the law. The responses were organized into thematic categories developed based on the main topics mentioned. Other binary variables (yes/no) in this section included: reports of visits by infant formula representatives to the health center (coordinators and professionals); distribution of gifts to health professionals; receipt of printed materials about infant formula delivered by representatives (professionals); receipt of financial support for participation in scientific events and courses (professionals); distribution of infant formula materials to mothers (professionals); reports on the distribution of these materials by professionals (coordinators); and contact between infant formula industry representatives and mothers (coordinators). The questions in this section were taken from the Training Course Manual on Monitoring by the International Baby Food Action Network Brazil (https://ibfan.org.br/publicacoes-ibfan). Additionally, compliance with Law No. 11,265/2006 was assessed through the researcher’s direct observation of the physical environment of the center for the presence of advertisements for infant formula and related childcare products.

The third section included questions about the implementation of breastfeeding promotion and management actions carried out by professionals (yes/no), and for those who answered “yes,” they were asked about the types of actions and the timing of their implementation. Subsequently, the responses were organized into thematic categories developed based on the most frequent answers (coordinators and professionals).

In the fourth section, questions about the knowledge and use of the Food and Nutrition Surveillance System (SISVAN) (yes/no) were included. For “yes” responses, participants were asked about their roles (coordinators and professionals), whether they completed data entry (yes/no) (coordinators and professionals), and the use of data for planning health promotion actions in the center (yes/no) (coordinators). The answers to the open-ended questions were grouped into categories based on the similarity of the answers.

Sample size

The sample size was calculated based on the total number of health professionals working in primary health care in 2021, according to the National Registry of Health Establishments (*Cadastro Nacional dos Estabelecimentos de Saúde*). A total of 474 primary health care professionals were identified, including 125 physicians, 125 nurses, 12 nutritionists, and 212 nursing technicians. Subsequently, the health sector of the respective health center where these professionals worked was identified. Within each sector, health centers were selected through cluster sampling. Subsequently, the selection of professionals was carried out by simple random sampling. The number of professionals was proportional to the size of each sector. Because the study variables were organized as binary, a prevalence of 50% was adopted to maximize the sample size, with a maximum error of 5% and a confidence level of 95%. The total sample size and the number of professionals per health sector were reached.

Statistical methods

Data on knowledge of Law No. 11,265/2006, its compliance in health centers, breastfeeding promotion and management actions, as well as knowledge of the Food and Nutrition Surveillance System (SISVAN) were described using relative frequency. The mean and standard deviation of the age of the professionals were calculated, and the comparison according to professional training was performed by analysis of variance (ANOVA). Due to the asymmetric distribution, the length of service in the public sector was estimated by median, with the interquartile deviation, and comparisons by professional training were performed using the Kruskal-Wallis method. Knowledge and compliance with Law No. 11,265/2006 by professionals in health establishments, breastfeeding management in the units, and professionals’ understanding of the Food and Nutrition Surveillance System (SISVAN) were compared according to the care model (primary health care centers/family health centers) and professional training, using the chi-square test with a significance level of 5%. Statistically significant differences between professional categories were identified using different letters (Tables 2 and 4). Statistical analyses were performed using Stata SE 14.0 software.

## Results

The sample consisted of 215 health professionals: 57 physicians, 56 nurses, 6 nutritionists, and 96 nursing technicians. A total of five primary health care centers and 44 family health care centers were visited. All center coordinators were nurses (n=29).

The average age of professionals was slightly higher among technicians. The median length of service in the public health system was higher among nurses and technicians. The majority of professionals who completed postgraduate studies were nurses and nutritionists (especially *lato sensu* specialization). Half of the nurses reported having participated in breastfeeding management courses. One-third of the coordinators reported having taken service management courses. Only 6.9% of coordinators and 4.3% of health professionals participated in the single workshop on the Brazilian Breastfeeding and Complementary Feeding Strategy (EAAB) held in the municipality in 2017 (Table 1).

**Table 1:** Demographic characteristics (age), professional activity in the public service, participation in breastfeeding training courses, and use of breastfeeding update materials by professional training. Uberlândia, 2022

		Professional training				
	Total	Doctor	Nurse	Nutritionist	Nursing technician	p-value
	Mean (standard deviation)					
Age (mean)	37.3 (±9.8)	34.1 (±10.2)	37.2 (±7.1)	31.2 (±9.8)	39.6 (±10.5)	<0.001
	Median (1st quartile; 3rd quartile)					
Time in the public health system (months)	69 (24; 144)	36 (9; 72)	84 (30; 156)	24 (20; 48)	102 (36; 174)	<0.001
	Relative frequency (%)					
Completion of residency/postgraduate studies	38.1	38.6	96.4	83.3	1.0	<0.001
Use of materials for breastfeeding update	50.4	63.2	66.7	83.3	36.5	0.001
Participation in service management courses	31.0	-	-	-	-	-
Participation in breastfeeding management courses	25.8	28.1	51.6	-	18.8	0.003
Participation in the Brazilian Breastfeeding and Complementary Feeding Strategy (EAAB) workshop	4.3	5.3	14.8	-	1.0	0.07

The use of materials for breastfeeding updates was higher among nutritionists and below 50,0% among technicians (Table 1). When asked about the use of materials for breastfeeding updates, 89.7% of coordinators and 50.4% of professionals reported using them. The internet was cited as a data source by 3.8% of coordinators and 35.1% of professionals, with the most frequently reported materials being those produced by the Municipal Health Department/Ministry of Health (50.0% of coordinators and 37.2% of professionals). In addition to training materials from the municipal health network, the National Network of Human Milk Banks (*Rede Brasileira de Banco de Leite Humano*, rBLH-BR), and conferences (53.8% of coordinators and 8.5% of professionals), sources included scientific articles (3.8% of coordinators and 2.1% of professionals), books (3.8% of coordinators and 1.1% of professionals), and materials from the Brazilian Associations of Pediatrics and Gynecology (7.7% of coordinators and 6.4% of professionals). Finally, no coordinators and only 4.3% of professionals who reported using materials for breastfeeding updates explicitly mentioned the Dietary Guidelines for Brazilian Children Under Two Years (data not shown in tables).

Only 12.4% of those surveyed reported knowing Law No. 11,265/2006. Of these, 30.4% correctly described its objectives, such as support from the Ministry of Health for the protection of exclusive breastfeeding, the prohibition of infant formula promotion in health centers and on television, regulation of sales, and the regulation of infant formula advertising. Only physicians reported receiving printed materials from infant formula representatives, and although low, the percentage was higher among professionals working in primary health care centers. Among center coordinators, 27.6% reported distributing infant formula materials to families. During direct observation of the visited units, no advertisements for infant formula or related childcare products were identified (data not shown in tables).

Breastfeeding management was carried out mainly by physicians and nurses in a similar manner, according to the care model, and was concentrated at the time of the neonatal heel prick test. Breastfeeding counseling, which is frequently provided, focuses on latch and baby positioning during feeding, explains the importance of breastfeeding, and involves feeding observation, with higher percentages among nurses (Table 2). The main breastfeeding promotion action was the conduct of educational groups during pregnancy (Table 3).

**Table 2:** Relative frequency (%) of breastfeeding management practices in primary health care centers by professional training and care model (health centers - UBS and family health centers - UBSF). Uberlândia, 2022

		Professional training					Health care model		
	Total	Doctor	Nurse	Nutritionist	Technician	p-value	UBS	UBSF	p-value
	(n=215)	(n=57)	(n=56)	(n=6)	(n=96)				
	Relative frequency (%)								
Timing of breastfeeding counseling									
Performance of the neonatal heel prick by the nursing team	76.0	66.7	92.6	66.7	78.1	0.061	75.0	76.6	0.856
10-day consultation conducted by the medical team	23.7	59.6^a^	11.1^b^	16.7^b^	7.3^b^	0.000	10.7	26.6	0.071
Prenatal and postnatal medical appointments	11.4	10.5	11.1	16.7	7.3	0.786	7.1	9.5	0.691
Counseling provided									
Observation of breastfeeding during the medical appointment	21.8	14.0^a^	55.5^b^	-	17.7^a^	0.000	14.3	22.8	0.313
Latch and positioning	65.0	73.7	85.2	33.3	67.7	0.058	64.3	72.2	0.398
Fissures	14.7	15.8	29.6	-	13.5	0.153	14.3	16.5	0.774
Mastitis	5.9	12.3	11.1	-	0.1	0.844	3.6	12.7	0.161
Breast milk expression	18.9	21.1	22.2	16.7	15.6	0.792	7.1	20.3	0.098
Human Milk Bank Service	6.4	12.3	11.1	-	2.1	0.054	3.6	7.0	0.501
Number of feedings/on-demand feeding	15.1	19.3	7.4	16.7	14.6	0.559	10.7	15.8	0.486
Breast emptying/breast switching	9.5	12.3	18.5	-	7.3	0.276	10.7	10.1	0.925
Importance of breastfeeding and/or exclusive breastfeeding	29.2	38.6^a^	40.7^a^	16.7^a^	20.8^b^	0.048	32.1	28.5	0.694
Discouraging the use of pacifiers and baby bottles	2.6	1.8	7.4	-	1.0	0.233	3.6	1.9	0.574
Unable to inform	5.4	7.0	0.0	-	14.6	0.084	21.4	7.6	0.022

Different letters (a, b) indicate statistical significance between professional categories (professional training)

**Table 3:** Relative frequency (%) of types of breastfeeding promotion actions carried out by health professionals by care model (health centers - UBS and family health centers - UBSF). Uberlândia, 2022

	Health care model			
	Total	UBS	UBSF	
Promotion actions	Relative frequency (%)			p-value
Groups of pregnant people	82.3	91.3	73.2	0.062
Individualized appointments	19.1	13.0	25.2	0.205
counseling during the neonatal heel prick	8.5	13.0	3.9	0.074
Waiting rooms	19.1	21.7	16.5	0.544
Childcare groups	19.2	30.4	7.9	0.002
Agosto Dourado (“Golden August” Campaign)	2.8	0.0	5.5	0.249

Nurses and nutritionists reported knowing the Food and Nutrition Surveillance System (SISVAN) more frequently and correctly describing its objectives. Data entry into the system is primarily performed by nutritionists, followed by nurses. Almost all coordinators reported entering data into the system, but only 27.6% of them use its information to develop actions (Table 4). Only 2.8% of professionals reported receiving any feedback on the data they enter into the Food and Nutrition Surveillance System (SISVAN) from center coordinators or municipal management.

**Table 4:** Relative Frequency (%) of knowledge and use of the Food and Nutrition Surveillance System (SISVAN) by health professionals and coordinators of primary health care centers by professional training and care model (health centers - UBS and family health centers - UBSF). Uberlândia, 2022

	Professional training						Health care model		
	Total	Doctor	Nurse	Nutritionist	Technician	p-value	UBS	UBSF	p-value
	(n=215)	(n=57)	(n=56)	(n=6)	(n=96)				
	Relative frequency (%)								
Knowledge of SISVAN									
Professionals	77.7	56.1^a^	88.9^b^	100.0^b^	65.6^a^	0.008	67.9	67.1	0.936
Coordinators	100.0	-	-	-	-	-	100.0	100.0	-
If yes, aware of the SISVAN function									
Professionals	45.7	29,8^a^	59.3^b^	66.7^b^	27.1^a^	0.005	32.1	34.2	0.834
Coordinators	86.2	-	-	-	-	-	-	15.4	0.464
If yes, describe the function as nutrition/food surveillance									
Professionals	16.5	15.8^a^	14.8^a^	33.3^a^	2.1^b^	0.003	3.6	10.1	0.267
Coordinators	20.0	-	-	-	-	-	-	22.7	0.356
If yes, describe the function as nutrition/food assessment									
Professionals	14.8	1.8^a^	14.8^b^	33.3^b^	9.4^a^	0.024	7.1	8.9	0.765
Coordinators	52.0	-	-	-	-	-	100.0	45.5	0.076
If yes, describe the function as generating indicators									
Professionals	4.6	5.3	11.1	-	2.1	0.205	-	5.1	0.224
Coordinators	28.0	-	-	-	-	-	-	31.8	0.250
**If yes, describe the function as related to the Family Income Transfer Program (*Programa Bolsa Família*, PBF)**									
Professionals	0.5	-	-	-	2.1	0.594	3.6	0.6	0.165
Coordinators	-	-	-	-	-	-	-	-	-
If yes, describe the function with other responses									
Professionals	10.4	7.0	22.2	-	12.5	0.179	17.9	10.8	0.284
Coordinators	8.0	-	-	-	-	-	-	9.1	0.586
Complete SISVAN data									
Professionals	19.3	1.8^a^	29.6^b^	50.0^b^	25.0^b^	0.000	32.1	17.1	0.063
Coordinators	89.7	-	-	-	-	-	100.0	88.5	0.534
Use of SISVAN data to support actions									
Coordinators	27.6	-	-	-	-	-	66.7	23.1	0.110

Different letters (a, b) indicate statistical significance between professional categories (professional training)

## Discussion

Our results indicate that knowledge of Law No. 11,265/2006 remains low among the professionals interviewed, even after nearly 20 years since the law’s enactment. Despite the lack of knowledge, no advertisements for infant formula or related products, nor the presence of industry representatives, were identified in the centers. Breastfeeding management focuses on counseling about proper latch and baby positioning, regardless of professional training, and is one of the topics addressed in specific consultations. Most coordinators and professionals reported knowing the Food and Nutrition Surveillance System (SISVAN) and its objectives, especially nutritionists and nurses. However, the use of information from the System for planning actions remains infrequent among coordinators.

Although the data were compared according to health care models, we emphasize interpreting these results with caution, as the sample was not designed to ensure such representativeness. Another possible limitation is self-reporting, with responses differing from what is practiced by professionals. However, we consider this risk to be low, as we identified several situations that were not in accordance with recommended practices, and data collection was conducted by an interviewer external to the health services, which tends to reduce the influence of institutional ties or organizational loyalty on the responses provided.

The concept and objectives of Law No. 11,265/2006 are addressed in the Brazilian Breastfeeding and Complementary Feeding Strategy (EAAB) ^18^. We observed a low percentage of professionals who correctly express their concepts and objectives. The receipt of materials for formulas was reported only by physicians, mainly in facilities outside primary health care. Unlike the hospital environment, where formulas can be distributed and prescribed, this practice is unusual in health centers, which may explain the absence of industry representatives. In hospitals from six Brazilian cities, half of the professionals reported receiving gifts from industry ^19^. A study conducted with professionals working in hospitals indicated greater knowledge of the law (54%) compared with our results, especially among those accredited as members of the Baby-Friendly Hospital Initiative ^19^. Knowledge of Law No. 11,265/2006, along with the Ten Steps to Successful Breastfeeding, is among the topics addressed by these teams. Training on the law provided to pharmacy professionals in drugstores reduced violations in these establishments ^20^. Although this population differs from that covered in the present study, we emphasize the importance of training and knowledge of the law for the protection of breastfeeding. 

Receiving group counseling and demonstrations of breastfeeding techniques from primary health care professionals are associated with breastfeeding practice ^21^. In the present study, most professionals reported providing counseling on management during the newborn’s first medical appointment, with an emphasis on correcting latch and positioning. This initial medical appointment constitutes one of the few systematized health care practices for the mother-infant dyad in the analyzed centers, whereas other counseling largely depends on spontaneous demand from families and the internal organization of the health teams. However, a previous study indicates that specific counseling on latch and positioning-fundamental to the technical management of breastfeeding and requiring clinical expertise and time from professionals-has been less frequently addressed than more general breastfeeding topics ^22^.

Breastfeeding observation is conducted more frequently by nurses, the professionals most involved in breastfeeding management. Observation of breastfeeding enables the identification of aspects needing improvement, as well as starting from the practical situation of the mother-infant dyad to provide support, according to the methodological principles of the Brazilian Breastfeeding and Complementary Feeding Strategy (EAAB). Furthermore, a significant portion of respondents reported being unable to specify how breastfeeding management is conducted in the unit, particularly professionals from primary health care centers. Possibly, providing mothers with counseling on breastfeeding management, addressing factors beyond latch and positioning, could reduce the prescription of infant formula due to issues related to insufficient weight gain in babies, as reported by primary health care coordinators.

Along with the significant proportion of professionals not utilizing materials for updating knowledge on breastfeeding, the low participation of coordinators and health professionals in training sessions on the Brazilian Breastfeeding and Complementary Feeding Strategy (EAAB) may explain the lack of counseling provided to mothers regarding mastitis, breast milk expression techniques, on-demand feeding, breast emptying, and the use of pacifiers and bottles. These topics are covered during the training of tutors responsible for capacity building within primary health care teams ^3^. One of the strengths of the Strategy lies in its foundation on continuous health education, grounded in a critical-reflective approach that considers the complexity of each team member’s professional practice ^18^. 

It is noteworthy that none of the coordinators and fewer than 5.0% of health professionals explicitly referenced the Dietary Guidelines for Brazilian Children Under Two Years ^23^ as a source for continuing education and professional development. A study conducted with physicians, nurses, and community health workers also identified insufficient knowledge of the content of the Dietary Guidelines, which impacted their practices and recommendations to mothers and children ^24^. 

The Food and Nutrition Surveillance System is utilized within the Brazilian Breastfeeding and Complementary Feeding Strategy (EAAB) to monitor infant feeding practices. However, despite the vast majority of coordinators interviewed reporting familiarity with the System and its objectives, only a small proportion reported completing its data entries and utilizing the information to support actions within health centers. Consequently, the data available within the System do not permit a comprehensive assessment of the population served, thereby compromising the strategic planning of interventions tailored to the population’s genuine needs. Furthermore, the variation in data entry according to professional category is unjustified, given that the task is not exclusive to any specific professional group. Rolim et al ^25^clarify that the lack of data analysis, recommendation for use, and effective implementation of the Food and Nutrition Surveillance System (SISVAN) by the responsible professionals can be attributed to excessive workload, particularly among nurses, as well as difficulties accessing the internet, insufficient maintenance of anthropometric equipment, and a shortage of trained personnel. Periodic training on this System can significantly enhance professionals’ learning, enabling the provision of continuous and comprehensive care to individuals ^26^.

The scope and representativeness of the sample are underscored as key strengths of the study. It was also possible to analyze both the management and protection of breastfeeding through the examination of data related to Law No. 11,265/2006, as well as the utilization of the Food and Nutrition Surveillance System (SISVAN) for population diagnosis. Given the comprehensive coverage of primary health care in the municipality, the situational diagnosis conducted can serve as a foundation for the necessary actions regarding the promotion, management, and protection of breastfeeding, as well as for raising awareness among management to encourage adherence to the Brazilian Breastfeeding and Complementary Feeding Strategy (EAAB). Furthermore, this study, together with others, reflects the current state of primary health care within the context of the Brazilian Breastfeeding and Complementary Feeding Strategy (EAAB), serving as a basis for the design of effective and targeted actions directed at health care professionals by the Brazilian government.

In conclusion, notable deficiencies were identified in both the promotion and protection of breastfeeding practices, as well as in the application of the Food and Nutrition Surveillance System (SISVAN) within primary health care in the municipality under study (Uberlândia, Minas Gerais). Although the majority of professionals and coordinators report familiarity with the system, its utilization as a tool for population-level diagnosis and decision-making support within health care centers remains limited. Breastfeeding management practices, although present, appear to be insufficiently systematized, predominantly focusing on the newborn’s initial consultation, with an emphasis on counseling regarding latch and infant positioning. These findings underscore the need to strengthen initiatives focused on continuous education, broaden adherence to the Brazilian Breastfeeding and Complementary Feeding Strategy (EAAB), and enhance the utilization of the Food and Nutrition Surveillance System (SISVAN). It is further recommended that future research explore innovative interprofessional intervention strategies to strengthen clinical practices in breastfeeding within the primary health care context across different regions of the country.

## References

[1] Brasil, Ministério da Saúde (2013). Estratégia Nacional para Promoção do Aleitamento Materno e Alimentação Complementar Saudável - Estratégia Amamenta e Alimenta Brasil.

[2] Brasil, Ministério da Saúde (2008). http://bvsms.saude.gov.br/bvs/publicacoes/rede_amamenta_brasil_primeiros_passos.pdf.

[3] Brasil, Ministério da Saúde (2010). ENPACS: Estratégia Nacional Para Alimentação Complementar Saudável: Caderno Do Tutor/Ministério da Saúde, Rede Internacional em Defesa do Direito de Amamentar - IBFAN Brasil.

[4] World Health Organization (1981). International Code of Marketing of Breast-Milk Substitutes.

[5] Brasil (2018). DECRETO N^o^ 9.579, DE 22 DE NOVEMBRO DE 2018. Consolida atos normativos editados pelo Poder Executivo federal que dispõem sobre a temática do lactente, da criança e do adolescente e do aprendiz, e sobre o Conselho Nacional dos Direitos da Criança e do Adolescente, o Fundo Nacional para a Criança e o Adolescente e os programas federais da criança e do adolescente, e dá outras providências.

[6] Brasil (2006). Lei n^o^ 11.265, de 3 de janeiro de 2006. Regulamenta a comercialização de alimentos para lactentes e crianças de primeira infância e também a de produtos de puericultura correlatos.

[7] Brasil, Ministério da Saúde, Secretaria de Atenção à Saúde, Departamento de Atenção Básica (2013). Política Nacional de Alimentação e Nutrição / Ministério da Saúde, Secretaria de Atenção à Saúde. Departamento de Atenção Básica.Básica. - 1. ed., 1. reimpr..

[8] Brasil, Ministério da Saúde (2015). PORTARIA N^o^ 1.130, DE 5 DE AGOSTO DE 2015. Institui a Política Nacional de Atenção Integral à Saúde da Criança (PNAISC) no âmbito do Sistema Único de Saúde (SUS).

[9] Nascimento FA do, Silva SA da, Jaime PC (2019). Cobertura da avaliação do consumo alimentar no Sistema de Vigilância Alimentar e Nutricional Brasileiro: 2008 a 2013. Rev Bras Epidemiol.

[10] Ricci JMS, Romito ALZ, Silva SA da, Carioca AAF, Lourenço BH (2023). Marcadores do consumo alimentar do Sisvan: tendência temporal da cobertura e integração com o e-SUS APS, 2015-2019. Ciênc Saúde Coletiva.

[11] Brockveld L de SM (2016). O desafio de capacitar profissionais da Atenção Básica, em aleitamento materno e alimentação complementar. Bol Inst Saúde - BIS.

[12] Tavares JS, Vieira D de S, Dias TKC, Tacla MTGM, Collet N, Reichert AP da S (2018). Logframe Model as analytical tool for the Brazilian Breastfeeding and Feeding Strategy. Rev Nutr.

[13] Venancio SI, de Araújo BC, Oliveira C de F (2022). Effective interventions for the promotion of breastfeeding and healthy complementary feeding in the context of Primary Health Care. Rev Paul Pediatr.

[14] Bortolini GA (2017). Avaliação da implementação da estratégia amamenta e alimenta brasil (EAAB).

[15] Secretaria de Governo e Comunicação/PMU (2022). Uberlândia tem a melhor atenção primária do Brasil na Saúde.

[16] Brasil, Ministério da Saúde Cobertura da Atenção primária (PNS 2020-2023). e-gestor atenção primária à saúde.

[17] INSTITUTO BRASILEIRO DE GEOGRAFIA E ESTATÍSTICA (IBGE) (2023). Cidades e Estados do Brasil (Cidades@).

[18] Brasil, Ministério da Saúde (2015). Secretaria de Atenção à Saúde. Estratégia Nacional para Promoção do Aleitamento Materno e Alimentação Complementar Saudável no Sistema Único de Saúde: manual de implementação / Ministério da Saúde, Secretaria de Atenção à Saúde.

[19] Velasco ACCF, Oliveira MIC de, Boccolini CS (2022). Assédio da indústria de alimentos infantis a profissionais de saúde em eventos científicos. Rev Saude Pública.

[20] Rodrigues GPN, Oliveira MIC de, Boccolini CS, Sally E de OF, Moraes JR de (2021). Avaliação do impacto de intervenção educativa em farmácias com promoção comercial de produtos que competem com o aleitamento materno. Cad Saúde Pública.

[21] Pereira RSV, Oliveira MIC de, Andrade CLT de, Brito A dos S (2010). Fatores associados ao aleitamento materno exclusivo: o papel do cuidado na atenção básica. Cad Saúde Pública.

[22] Alves JS, Oliveira MIC, Rito RVVF (2018). Orientações sobre amamentação na atenção básica de saúde e associação com o aleitamento materno exclusivo. Cien Saude Colet.

[23] Brasil, Ministério da Saúde (2019). Guia alimentar para crianças menores de 2 anos.

[24] Amaral VL, Spadotto GC, Gomes CB (2024). Conhecimento, atitudes e práticas de profissionais da atenção primária sobre o guia alimentar para crianças até 2 anos: estudo transversal, Botucatu, São Paulo, 2023. Epidemiol Serv Saúde.

[25] Rolim MD, Lima SML, Barros DC de, Andrade CLT de (2015). Avaliação do SISVAN na gestão de ações de alimentação e nutrição em Minas Gerais, Brasil. Cien Saude Colet.

[26] Araujo e Silva LB, Rezende FAC, Schott E, Da Silva CA (2016). Capacitação de agentes comunitários de saúde para o fortalecimento do SISVAN. Rev Ciênc Ext.

