# Supplementary material for: Knowledge and actions for the promotion and protection of breastfeeding by primary health care professionals in Uberlândia, 2022
Source: Epidemiol Serv Saude. 2026 Feb 16;35:e20240245. doi: 10.1590/S2237-96222026v35e20240245.en (PMC12917949; doi:10.1590/S2237-96222026v35e20240245.en)
Supplement: Translation

# Translation: Conhecimento e ações de promoção e proteção ao aleitamento materno por profissionais da atenção primária à saúde em Uberlândia, 2022

**DOI:** 10.1590/S2237-96222026v35e20240245.pt

**Authors:** Amanda Borges Amaral, Vivian Mara Gonçalves de Oliveira Azeredo, Leandro Alves Pereira, Ana Elisa Madalena Rinaldi

**Affiliations:** 1 Universidade Federal de Uberlândia, Faculdade de Medicina, Uberlândia, MG, Brazil; 2 Universidade Federal de Uberlândia, Faculdade de Educação Física e Fisioterapia, Uberlândia, MG, Brazil; 3 Universidade Federal de Uberlândia, Instituto de Matemática e Estatística, Uberlândia, MG, Brazil

**Keywords:** Aleitamento Materno, Atenção Primária à Saúde, Aplicação da Lei, Vigilância Alimentar e Nutricional, Estudo Transversal

## Abstract

**Objetivo:**

Analisar, no âmbito da atenção primária, ações de promoção e de proteção ao aleitamento materno, o conhecimento e o cumprimento da Lei n° 11.265/2006 e o uso do Sistema Nacional de Vigilância Alimentar e Nutricional pelos profissionais da saúde.

**Métodos:**

Estudo transversal, com amostra probabilística de coordenadores e de profissionais da saúde. Dois questionários foram elaborados para coleta de dados sobre formação profissional: conhecimento e cumprimento da Lei nº 11.265/2006; e conhecimento e utilização do Sistema de Vigilância Alimentar e Nutricional. Os dados foram expressos em frequência relativa e utilizou-se teste qui-quadrado para compará-los segundo modelo de atenção e formação profissional.

**Resultados:**

Participaram do estudo 215 profissionais da saúde; apenas 12,4% profissionais afirmaram conhecer a Lei nº 11.265/2006, e 30,4% profissionais descreveram seus objetivos. Realização de grupos de gestantes foi a principal ação de promoção do aleitamento materno (82,3%), e a maioria dos profissionais (76,0%) realizava o seu manejo, focando na pega e na posição. Todos os coordenadores e 77,7% dos profissionais afirmaram conhecer o Sistema de Vigilância Alimentar e Nutricional, enquanto 86,2% dos coordenadores e 45,7% dos profissionais sabiam descrever seu objetivo; somente 27,6% dos coordenadores utilizavam seus dados para elaborarem ações na unidade.

**Conclusão:**

A maioria dos profissionais desconhece a principal lei de proteção ao aleitamento materno. Grupos de gestantes constituem a principal ação de promoção ao aleitamento materno. Apesar do conhecimento sobre o Sistema de Vigilância Alimentar e Nutricional, seu uso para o embasamento de ações é insuficiente.

Aspectos éticos

Esta pesquisa respeitou os princípios éticos, obtendo os seguintes dados de aprovação:

Comitê de ética em pesquisa: Universidade Federal de Uberlândia

Número do parecer: 4.910.277

Data de aprovação: 16/8/2021

Certificado de apresentação de apreciação ética: 50201321.0.0000.5152

Termo de consentimento livre e esclarecido: Obtido de todos os participantes antes da coleta.

## Introdução

No Brasil, desde 2007, foram elaboradas três estratégias voltadas à qualificação dos profissionais da atenção primária para manejo da alimentação infantil (1, 2, 3). Tais estratégias visam aprimorar os processos de trabalho por meio da educação crítico-reflexiva, com ênfase na formação de tutores e na realização de oficinas de trabalho. A Rede Amamenta Brasil tem como objetivo qualificar profissionais da saúde para a promoção e o manejo do aleitamento materno (2). Complementarmente, em 2010, foi proposta a Estratégia Nacional para Promoção da Alimentação Complementar Saudável, como instrumento para apoiar e fortalecer ações voltadas à alimentação complementar, especialmente na atenção básica (3). Em 2013, ambas foram unificadas na Estratégia Amamenta e Alimenta Brasil (1), visando à promoção e à proteção do aleitamento materno e da alimentação complementar.

A certificação das equipes da atenção primária na Estratégia Amamenta e Alimenta Brasil baseia-se no monitoramento de indicadores de aleitamento materno e de alimentação complementar e no cumprimento da Norma Brasileira de Comercialização de Alimentos para Lactentes e Crianças de primeira infância, bicos, chupetas e mamadeiras (1). Essa norma foi criada a partir do Código Internacional de Comercialização de Substitutos do Leite Materno, para regulamentar a atividade de marketing das indústrias de alimentos infantis e de produtos correlatos (4). Foi aprovada como Lei nº 11.265 em 2006 e regulamentada pelo Decreto nº 9.579 em 2018, com o objetivo de proteger o aleitamento materno (5, 6).

O monitoramento dos indicadores de aleitamento materno e de alimentação complementar deve ser realizado a partir do Sistema de Vigilância Alimentar e Nutricional, conforme diretrizes da Política Nacional de Alimentação e Nutrição (7) e da Política Nacional de Atenção Integral à Saúde da Criança ( 8). Apesar dos avanços no registro dos marcadores de consumo alimentar entre 2008 a 2019, a cobertura e o uso do sistema ainda são limitados. Entre 2008 e 2013 (9), a cobertura variou de 0,1% a 0,4%, e, entre 2015 e 2019 (10), de 0,2% a 0,9%, com diferenças regionais e maior cobertura entre crianças menores de quatro anos.

A implementação da Estratégia Amamenta e Alimenta Brasil não é homogênea entre os municípios brasileiros (11, 12). Até 2019, cerca de 48 mil profissionais da saúde (13) participaram de capacitações, entretanto a estratégia está consolidada apenas em 9,4% das unidades de saúde do país, com maior abrangência nas regiões Centro-Oeste e Norte (14). 

Minas Gerais destacou-se pelo maior número de oficinas realizadas e tutores formados (14). Em Uberlândia, Minas Gerais, foi realizada somente uma oficina em 2017. Em 2022, a cidade obteve o melhor desempenho no índice sintético final do Ministério da Saúde entre os municípios com mais de 500 mil habitantes (15, 16). Diante dessa abrangente cobertura, torna-se necessário analisar detalhadamente as ações de promoção e de proteção ao aleitamento materno na atenção primária, as recomendações que orientam as condutas profissionais e o conhecimento dos profissionais sobre componentes essenciais da Estratégia Amamenta e Alimenta Brasil. Assim, objetivou-se analisar, no âmbito da atenção primária, ações de promoção e de proteção ao aleitamento materno, o conhecimento e o cumprimento da Lei nº 11.265/2006, bem como o conhecimento e o uso do Sistema Nacional de Vigilância Alimentar e Nutricional pelos profissionais da saúde.

## Métodos

Delineamento do estudo e contexto

Tratou-se de estudo transversal realizado nas unidades básicas de saúde e nas unidades básicas de saúde da família em Uberlândia, Minas Gerais, no período de janeiro a julho de 2022. A população do município é de 713.222 habitantes (17) e, no ano da coleta de dados, a cobertura da atenção primária foi de 93,4% (16). Em 2022, existiam no município oito unidades básicas de saúde e 58 unidades básicas de saúde da família, distribuídas em cinco setores de saúde (Norte/Sul/Leste/Oeste/Central).

Participantes

O público participante incluiu profissionais da saúde que atendem na atenção primária e que prestam assistência a crianças menores de 2 anos e/ou a gestantes, sendo constituído de médicos/as, enfermeiros/as, nutricionistas, técnicos/as de enfermagem, bem como os/as profissionais que estavam, no momento da pesquisa, atuando como coordenadores/as da unidade.

Variáveis e mensuração dos dados

Foram elaborados dois questionários especificamente para este estudo, organizados em quatro seções, sendo um direcionado aos profissionais de saúde e outro aos coordenadores das unidades. A opção por questões abertas se justifica pelos dois objetivos centrais do estudo: o conhecimento da Lei nº 11.265/2006 e do Sistema Nacional de Vigilância Alimentar e Nutricional, e a realização das ações de promoção e de proteção ao aleitamento materno praticadas pelos profissionais da saúde. As questões abertas nos auxiliaram em não indução da resposta dos participantes. Os dois questionários foram testados em um estudo piloto, realizado em uma unidade básica de saúde em 2021, para conhecimento do tempo de aplicação e da conformidade das respostas dos profissionais às perguntas realizadas. Para cada questão do questionário, foi descrito o que se esperava de resposta. Quando as respostas dos profissionais não se alinhavam às respostas esperadas, a pergunta foi modificada a fim de se obter o conteúdo esperado. O estudo piloto e a coleta de dados foram realizados por uma única pesquisadora, visando à padronização da coleta de dados e a oportunidade para conhecer a estrutura física das unidades; uma das questões do questionário incluiu a observação direta de possíveis propagandas de fórmulas infantis e de produtos correlatos. No momento da digitação e da análise dos dados, foi investigada a consistência das respostas. Não houve respostas ausentes nos questionários. Na primeira seção do questionário, foram incluídos dados demográficos (idade), de formação (profissão e realização de pós-graduação lato sensu [especialização, residência]) (sim/não) e de atuação profissional para coordenadores das unidades e profissionais - tempo de trabalho na rede pública (meses), uso de material para atualização em aleitamento materno (sim/não), participação em cursos sobre manejo do aleitamento materno (sim/não - somente para profissionais de saúde), participação em cursos de gestão em serviços (sim/não - somente para coordenadores de unidades) e participação em oficinas da capacitação da Estratégia Amamenta e Alimenta Brasil (sim/não).

Na segunda seção, foram incluídas questões sobre o conhecimento da Lei nº 11.265/2006 e o seu cumprimento nas unidades de saúde (sim/não). Para os profissionais que responderam “sim”, foi perguntado se conheciam os objetivos da lei (questão aberta). As respostas foram organizadas em categorias temáticas elaboradas a partir dos temas principais citados. Outras variáveis binárias (sim/não) compuseram essa seção: relato de visita de representantes de fórmulas infantis na unidade de saúde (coordenadores e profissionais); entrega de brindes para o profissional de saúde; recebimento de material impresso sobre fórmula infantil entregue por representantes (profissionais); recebimento de apoio financeiro para participação em eventos científicos e cursos (profissionais); entrega de materiais sobre fórmula infantil às mães (profissionais) e relato sobre a entrega desses materiais pelos profissionais (coordenadores); contato entre representantes das indústrias de fórmula infantil e as mães (coordenadores). As questões dessa seção foram retiradas do Manual do Curso de Capacitação em Monitoramento da International Baby Food Action Network Brasil (https://ibfan.org.br/publicacoes-ibfan). Adicionalmente, o cumprimento da Lei nº 11.265/2006 foi analisado pela observação direta do ambiente físico da unidade pela pesquisadora sobre a presença de propaganda de fórmula infantil e de produtos de puericultura correlatos.

Na terceira seção, foram incluídas questões sobre a realização de ações de promoção e de manejo do aleitamento materno empreendidas pelos profissionais (sim/não), e, para os que responderam “sim”, foram questionados os tipos de ações e o momento de realização. Posteriormente, as respostas foram organizadas em categorias temáticas elaboradas a partir das respostas com maior frequência (coordenadores e profissionais).

Na quarta seção, foram incluídas questões sobre o conhecimento e o uso do Sistema de Vigilância Alimentar e Nutricional (sim/não). Para respostas “sim”, foram questionadas as suas funções (coordenadores e profissionais), a realização de preenchimento (sim/não) (coordenadores e profissionais) e o uso dos dados para o planejamento de ações de promoção à saúde na unidade (sim/não) (coordenadores). As respostas das questões abertas foram agrupadas em categorias com base na similaridade das respostas.

Tamanho da amostra

O cálculo da amostra foi realizado a partir do número total de profissionais da saúde que atuavam na atenção primária em 2021, segundo o Cadastro Nacional dos Estabelecimentos de Saúde. Foram identificados 474 profissionais da atenção primária, sendo 125 médicos, 125 enfermeiros, 12 nutricionistas e 212 técnicos de enfermagem. Posteriormente, foi identificado o setor de saúde da respectiva unidade de saúde que esses profissionais atuavam. Dentro de cada setor, foram selecionadas as unidades de saúde por amostragem por conglomerado. Posteriormente, a seleção dos profissionais foi realizada por amostragem aleatória simples. O número de profissionais foi proporcional ao tamanho em cada setor. Em função das variáveis do estudo serem organizadas como binárias, adotou-se a prevalência de 50%, com o objetivo de maximizar a amostra, erro máximo de 5% e nível de confiança de 95%. O número amostral total e o número de profissionais por setor de saúde foram alcançados.

Métodos estatísticos

Os dados sobre o conhecimento da Lei nº 11.265/2006, o seu cumprimento nas unidades de saúde, as ações de promoção e de manejo do aleitamento materno, bem como o conhecimento do Sistema de Vigilância Alimentar e Nutricional foram descritos em frequência relativa. Foram calculados a média e o desvio-padrão da idade dos profissionais, e a comparação segundo a formação profissional foi realizada pela análise de variância (ANOVA). Em função da distribuição assimétrica, o tempo de atuação no serviço público foi estimado em mediana, e o desvio interquartílico e a comparação segundo formação profissional foram realizados pelo método de Kruskal-Wallis. O conhecimento e o cumprimento da Lei nº 11.265/2006 pelos profissionais nos estabelecimentos de saúde, o manejo do aleitamento materno nas unidades e o entendimento dos profissionais sobre o Sistema de Vigilância Alimentar e Nutricional foram comparados segundo o modelo de atenção (unidades básicas de saúde/unidades básicas de saúde da família) e a formação profissional, usando-se o Teste de qui-quadrado e adotando-se o nível de significância de 5%. As diferenças com significância estatística entre as categorias profissionais foram identificadas com letras diferentes (Tabelas 2 e 4). As análises estatísticas foram realizadas no programa Stata SE 14.0.

## Resultados

A amostra foi composta por 215 profissionais da saúde, sendo 57 médicos, 56 enfermeiros, seis nutricionistas e 96 técnicos de enfermagem. No total, foram visitadas cinco unidades básicas e 44 unidades básicas de saúde da família. Todas as coordenadoras das unidades eram enfermeiras (n=29).

A média de idade dos profissionais foi ligeiramente superior entre técnicos. O tempo mediano de atuação na rede pública foi superior entre enfermeiros e técnicos. A maioria dos profissionais que realizaram pós-graduação foi de enfermeiros e de nutricionistas (especialmente, especialização lato sensu). Metade dos enfermeiros relatou ter participado de cursos de manejo do aleitamento materno. Um terço dos coordenadores relatou ter feito curso de gestão de serviços. Apenas 6,9% dos coordenadores e 4,3% dos profissionais de saúde participaram da única oficina sobre a Estratégia Amamenta e Alimenta Brasil realizada no município em 2017 (Tabela 1).

**Tabela 1:** Caracterização demográfica (idade), atuação profissional no serviço público, participação em cursos de capacitação do aleitamento materno e uso de materiais atualização em aleitamento materno segundo formação profissional. Uberlândia, 2022

		Formação profissional				
	Total	Médico	Enfermeiro	Nutricionista	Técnico em enfermagem	p-valor
	Média (desvio-padrão)					
Idade (média)	37,3 (±9,8)	34,1 (±10,2)	37,2 (±7,1)	31,2(±9,8)	39,6 (±10,5)	<0,001
	Mediana (1º quartil; 3º quartil)					
Tempo de atuação na rede pública (meses)	69 (24; 144)	36 (9; 72)	84 (30; 156)	24 (20; 48)	102 (36; 174)	<0,001
	Frequência relativa (%)					
Realização de residência/pós-graduação	38,1	38,6	96,4	83,3	1	<0,001
Uso de material para atualização em aleitamento materno	50,4	63,2	66,7	83,3	36,5	0,001
Participação em cursos de gestão em serviços	31	-	-	-	-	
Participação em cursos de manejo do aleitamento materno	25,8	28,1	51,6	-	18,8	0,003
Participação na oficina da Estratégia Amamenta e Alimenta Brasil	4,3	5,3	14,8	-	1	0,07

O uso de materiais para atualização em aleitamento materno foi superior entre nutricionistas e inferior a 50,0% entre os técnicos (Tabela 1). Quando questionados sobre o uso de materiais para atualização sobre aleitamento materno, 89,7% dos coordenadores e 50,4% dos profissionais afirmaram utilizá-los. A internet foi citada como fonte de dados por 3,8% dos coordenadores e 35,1% dos profissionais, e os materiais mais relatados foram aqueles elaborados pela Secretaria Municipal de Saúde/Ministério da Saúde (50,0% dos coordenadores e 37,2% dos profissionais). Além de materiais de capacitações da rede municipal de saúde, da Rede Brasileira de Banco de Leite Humano e de congressos (53,8% dos coordenadores e 8,5% dos profissionais), artigos científicos (3,8% dos coordenadores e 2,1% dos profissionais), livros (3,8% dos coordenadores e 1,1% dos profissionais) e materiais das Sociedades Brasileiras de Pediatria e Ginecologia (7,7% dos coordenadores e 6,4% dos profissionais). Por fim, nenhum coordenador e apenas 4,3% dos profissionais que afirmaram utilizar material para atualização em aleitamento materno citaram explicitamente o Guia Alimentar para Crianças Brasileiras Menores de Dois Anos (dados não mostrados em tabelas).

Apenas 12,4% dos investigados afirmaram conhecer a Lei nº 11.265/2006 e, desses, 30,4% descreveram de forma correta seus objetivos, tais como o apoio do Ministério da Saúde para proteção do aleitamento materno exclusivo, a proibição de indicação de fórmula infantil nas unidades de saúde e em televisão, a normatização da venda e a regulamentação de propagandas de fórmula infantil. Apenas os médicos afirmaram receber material impresso dos representantes de fórmula infantil e, embora baixo, o percentual foi superior entre os profissionais que atuavam em unidade básica de saúde. Entre os coordenadores das unidades, 27,6% afirmaram entregar material sobre fórmula infantil às famílias. Na observação direta das unidades visitadas, não foi identificada propaganda de fórmula infantil e de produtos de puericultura correlatos (dados não apresentados em tabelas).

O manejo do aleitamento materno foi realizado especialmente por médicos e por enfermeiros de forma semelhante, segundo modelo de atenção, e concentrou-se no momento de realização do teste do pezinho. As orientações sobre o aleitamento materno, realizadas frequentemente, focaram na pega e na posição do bebê na mamada, na descrição da importância do aleitamento materno e na observação da mamada, com maiores percentuais entre os enfermeiros (Tabela 2). A principal ação de promoção do aleitamento materno foi a realização de grupos educativos durante a gestação (Tabela 3).

**Tabela 2:** Frequência relativa (%) da realização do manejo do aleitamento materno nas unidades básicas de saúde segundo formação profissional e modelo de atenção (unidade básica de saúde - UBS e unidade básica de saúde da família - UBSF). Uberlândia, 2022

		Formação profissional					Modelo de atenção		
	Total	Médico	Enfermeiro	Nutricionista	Técnico	p-valor	UBS	UBSF	p-valor
	(n=215)	(n=57)	(n=56)	(n=6)	(n=96)				
	Frequência relativa (%)								
Momento de realização das orientações do aleitamento materno									
Realização do teste do pezinho pela equipe de enfermagem	76	66,7	92,6	66,7	78,1	0,061	75	76,6	0,856
Consulta de 10 dias realizada pela equipe médica	23,7	59,6^a^	11,1^b^	16,7^b^	7,3^b^	0	10,7	26,6	0,071
Consultas pré-natal e pós-natal	11,4	10,5	11,1	16,7	7,3	0,786	7,1	9,5	0,691
Orientações realizadas									
Observação da mamada durante consulta	21,8	14,0^a^	55,5^b^	-	17,7^a^	0	14,3	22,8	0,313
Pega e posição	65	73,7	85,2	33,3	67,7	0,058	64,3	72,2	0,398
Fissuras	14,7	15,8	29,6	-	13,5	0,153	14,3	16,5	0,774
Mastite	5,9	12,3	11,1	-	0,1	0,844	3,6	12,7	0,161
Extração do leite materno	18,9	21,1	22,2	16,7	15,6	0,792	7,1	20,3	0,098
Serviço do Banco de Leite Humano	6,4	12,3	11,1	-	2,1	0,054	3,6	7	0,501
Número de mamadas/livre demanda	15,1	19,3	7,4	16,7	14,6	0,559	10,7	15,8	0,486
Esvaziamento de mamas/troca de mamas	9,5	12,3	18,5	-	7,3	0,276	10,7	10,1	0,925
Importância do aleitamento materno e/ou AME	29,2	38,6^a^	40,7^a^	16,7^a^	20,8^b^	0,048	32,1	28,5	0,694
Desencorajamento do uso de chupetas e mamadeiras	2,6	1,8	7,4	-	1	0,233	3,6	1,9	0,574
Não sabe informar	5,4	7	0	-	14,6	0,084	21,4	7,6	0,022

Letras diferentes (a, b) indicam significância estatística entre as categorias profissionais (formação profissional).

**Tabela 3:** Frequência relativa (%) dos tipos de ações de promoção do aleitamento materno realizadas pelos profissionais de saúde segundo modelo de atenção (unidade básica de saúde - UBS e unidade básica de saúde da família - UBSF). Uberlândia, 2022

		Modelo de atenção		
	Total (n=215)	UBS	UBSF	
Ações de promoção	Frequência relativa (%)			p-valor
Grupos de gestantes	82,3	91,3	73,2	0,062
Consultas individualizadas	19,1	13	25,2	0,205
Orientações no teste do pezinho	8,5	13	3,9	0,074
Salas de espera	19,1	21,7	16,5	0,544
Grupos de puericultura	19,2	30,4	7,9	0,002
Agosto dourado	2,8	0	5,5	0,249

Enfermeiros e nutricionistas relataram com maior frequência conhecer o Sistema de Vigilância Alimentar e Nutricional e descrever corretamente os seus objetivos. O preenchimento dos dados no sistema é realizado principalmente por nutricionistas, seguido pelos enfermeiros. Quase todas as coordenadoras relataram preencher o sistema, mas somente 27,6% delas utilizam suas informações para elaborar ações (Tabela 4). Apenas 2,8% dos profissionais informaram receber algum retorno sobre os dados que preenchem pelo Sistema de Vigilância Alimentar e Nutricional dos coordenadores da unidade de saúde ou da gestão municipal.

**Tabela 4:** Frequência relativa (%) do conhecimento e uso do Sistema de Vigilância Alimentar e Nutricional (SISVAN) pelos profissionais de saúde e coordenadores das unidades básicas de saúde segundo formação profissional e modelo de atenção (unidade básica de saúde - UBS e unidade básica de saúde da família - UBSF). Uberlândia, 2022

		Formação profissional					Modelo de atenção		
	Total	Médico	Enfermeiro	Nutricionista	Técnico	p-valor	UBS	UBSF	p-valor
	(n=215)	(n=57)	(n=56)	(n=6)	(n=96)				
	Frequência relativa (%)								
Conhecimento do SISVAN									
Profissionais	77,7	56,1^a^	88,9^b^	100,0^b^	65,6^a^	0,008	67,9	67,1	0,936
Coordenadores	100	-	-	-	-	-	100	100	-
Se sim, conhece a função do SISVAN									
Profissionais	45,7	29,8^a^	59,3^b^	66,7^b^	27,1^a^	0,005	32,1	34,2	0,834
Coordenadores	86,2	-	-	-	-	-	-	15,4	0,464
Se sim, descrição da função como vigilância alimentar/nutricional									
Profissionais	16,5	15,8^a^	14,8^a^	33,3^a^	2,1^b^	0,003	3,6	10,1	0,267
Coordenadores	20	-	-	-	-	-	-	22,7	0,356
Se sim, descrição da função como avaliação alimentar/nutricional									
Profissionais	14,8	1,8^a^	14,8^b^	33,3^b^	9,4^a^	0,024	7,1	8,9	0,765
Coordenadores	52	-	-	-	-	-	100	45,5	0,076
Se sim, descrição da função como gerar indicadores									
Profissionais	4,6	5,3	11,1	-	2,1	0,205	-	5,1	0,224
Coordenadores	28	-	-	-	-	-	-	31,8	0,25
Se sim, descrição da função como relação com o Bolsa Família									
Profissionais	0,5	-	-	-	2,1	0,594	3,6	0,6	0,165
Coordenadores	-	-	-	-	-				
Se sim, descrição da função com outras respostas									
Profissionais	10,4	7	22,2	-	12,5	0,179	17,9	10,8	0,284
Coordenadores	8	-	-	-	-	-	-	9,1	0,586
Realiza o preenchimento do SISVAN									
Profissionais	19,3	1,8^a^	29,6^b^	50,0^b^	25,0^b^	0	32,1	17,1	0,063
Coordenadores	89,7	-	-	-	-	-	100	88,5	0,534
Utilização das respostas do SISVAN para embasamento de ações									
Coordenadores	27,6	-	-	-	-	-	66,7	23,1	0,11

Letras diferentes (a, b) indicam significância estatística entre as categorias profissionais (formação profissional).

## Discussão

Nossos resultados sinalizam que o conhecimento da Lei nº 11.265/2006 permanece baixo entre os profissionais entrevistados, mesmo após quase 20 anos de existência da lei. Apesar do desconhecimento, não foram identificadas propagandas de fórmula infantil ou produtos correlatos, nem a presença de representantes de indústrias nas unidades. O manejo do aleitamento materno concentra-se em orientações sobre a pega correta e o posicionamento do bebê, independentemente da formação profissional, sendo um dos temas abordados em consultas específicas. A maioria dos coordenadores e dos profissionais afirmou conhecer o Sistema de Vigilância Alimentar e Nutricional e os seus objetivos, especialmente nutricionistas e enfermeiros. Entretanto, o uso da informação do Sistema para planejamento das ações permanece pouco frequente entre os coordenadores.

Apesar da comparação dos dados segundo modelos de atenção à saúde, destacamos a interpretação desses resultados com cautela, pois a amostra não foi desenhada para garantir essa representatividade. Outra possível limitação é o autorrelato, com respostas diferentes do que é realizado na prática dos profissionais. Entretanto, julgamos que esse risco seja baixo, pois identificamos diversas situações desconformes com as práticas recomendadas, e a coleta dos dados foi realizada por entrevistadora externa aos serviços de saúde, o que tende a reduzir a influência de vínculos institucionais ou de lealdade organizacional sobre as respostas fornecidas.

O conceito e os objetivos da Lei nº 11.265/2006 são discutidos na Estratégia Amamenta e Alimenta Brasil (18). Observamos baixo percentual de profissionais que expressam corretamente seus conceitos e objetivos. O recebimento de materiais sobre fórmulas foi relatado apenas por médicos, principalmente em estabelecimentos fora da atenção primária. Diferentemente do ambiente hospitalar, onde fórmulas podem ser distribuídas e prescritas, essa prática é incomum nas unidades de saúde, o que pode explicar a ausência de representantes da indústria. Em hospitais de seis cidades brasileiras, metade dos profissionais relatou receber brindes das indústrias (19). Estudo realizado com profissionais que trabalham em hospitais indicou maior conhecimento da lei (54,0%) em comparação aos nossos resultados, especialmente entre os credenciados como Hospitais Amigos da Criança (19). O conhecimento da Lei nº 11.265/2006, junto com os 10 passos para o sucesso do aleitamento materno, integra os temas trabalhados por essas equipes. A capacitação sobre a lei voltada aos farmacêuticos de drogarias reduziu as infrações nesses estabelecimentos (20). Embora esse público seja distinto do abrangido neste estudo, destacamos a importância da capacitação e do conhecimento da lei para a proteção do aleitamento materno.

O recebimento de orientações em grupo e da demonstração de técnicas de amamentação por profissionais da atenção primária estão associados à prática do aleitamento materno (21). Neste estudo, a maioria dos profissionais relatou realizar orientações sobre manejo na primeira consulta do recém-nascido, com ênfase na correção da pega e da posição. Essa consulta inicial configura uma das poucas práticas sistematizadas de cuidado ao binômio mãe-bebê nas unidades analisadas, enquanto demais orientações dependem, em grande parte, da demanda espontânea das famílias e da organização interna das equipes de saúde. Entretanto, estudo anterior aponta que orientações específicas sobre a pega e a posição - fundamentais ao manejo técnico da amamentação e que exigem tempo e competência clínica dos profissionais - têm sido menos abordadas que conteúdos generalistas sobre a amamentação (22).

A observação da mamada é realizada com maior frequência por enfermeiros, profissionais mais envolvidos no manejo do aleitamento materno. A observação da mamada permite a identificação de pontos a serem melhorados, bem como partir de situação prática da díade para o seu auxílio, conforme pressupostos metodológicos da Estratégia Amamenta e Alimenta Brasil. Além disso, parte expressiva dos entrevistados relatou não saber informar como é feito o manejo do aleitamento materno na unidade, especialmente os profissionais das unidades básicas de saúde. Possivelmente, a orientação das mães sobre o manejo do aleitamento materno para fatores além da pega e da posição diminuiria a prescrição de fórmula infantil por fatores associados ao ganho de peso insuficiente dos bebês relatado pelos coordenadores da atenção primária.

A baixa participação de coordenadores e de profissionais em capacitações sobre a Estratégia Amamenta e Alimenta Brasil e o percentual expressivo de profissionais que não utilizam material para a atualização em aleitamento materno pode justificar a falta de orientação às mães sobre mastite, forma de ordenha, livre demanda, esvaziamento da mama e uso de chupetas e mamadeiras, visto que esses temas são abordados na formação de tutores com a função de capacitar a equipe na atenção primária (3). Um dos pontos positivos da Estratégia é ser baseada na educação permanente em saúde, ancorada na concepção crítico-reflexiva, partindo da complexidade da atuação profissional de cada membro da equipe (18).

Destacamos que nenhum coordenador e que menos de 5,0% dos profissionais citaram explicitamente o Guia Alimentar para Crianças Brasileiras Menores de Dois Anos (23) como material de atualização. Estudo realizado com médicos, enfermeiros e agentes comunitários de saúde também identificaram conhecimento insuficiente do conteúdo do guia alimentar, repercutindo nas práticas e nas recomendações às mães e às crianças (24).

O Sistema de Vigilância Alimentar e Nutricional é utilizado na Estratégia Amamenta e Alimenta Brasil para fazer o monitoramento das práticas da alimentação infantil. Porém, apesar de a grande maioria dos coordenadores entrevistados relatarem conhecer o Sistema e seus objetivos, pequena proporção relatou fazer seu preenchimento e utilizar as informações para embasar ações nas unidades de saúde. Dessa forma, os dados disponíveis no Sistema não possibilitam o diagnóstico da população atendida, implicando na dificuldade de planejamento de ações focadas em reais necessidades da população. Além disso, a variação do preenchimento segundo formação profissional não se justifica uma vez que ele não é específico de uma categoria profissional. Rolim et al (25) esclarecem que a falta de análise de dados, de recomendação do uso e de execução do Sistema de Vigilância Alimentar e Nutricional pelos profissionais responsáveis pode ser justificado pelo excesso de trabalho dos profissionais, especialmente do enfermeiro, além de dificuldade de acesso à internet e falta de manutenção de equipamentos antropométricos e de profissionais capacitados. Capacitações periódicas sobre esse Sistema podem promover melhora significativa no aprendizado dos profissionais, possibilitando que o atendimento prestado ao indivíduo seja contínuo e completo (26).

Destacamos como pontos fortes do estudo a abrangência e a representatividade da amostra. Também foi possível analisar tanto o manejo do aleitamento materno e da sua proteção, através da exploração dos dados referentes à Lei nº 11.265/2006, quanto a utilização do Sistema de Vigilância Alimentar e Nutricional para diagnóstico populacional. Pela cobertura abrangente da atenção primária no município, o diagnóstico situacional realizado pode embasar ações necessárias a serem realizadas no que tange à promoção, ao manejo e à proteção do aleitamento materno, além da sensibilização da gestão para adesão à Estratégia Amamenta e Alimenta Brasil. Além disso, este estudo, em conjunto com outros, reflete a situação da atenção primária no contexto da Estratégia Amamenta e Alimenta Brasil, servindo de base para o delineamento de ações efetivas e direcionadas aos profissionais pelo governo brasileiro.

Conclui-se que há fragilidades importantes nas ações de promoção e de proteção ao aleitamento materno, bem como no uso do Sistema de Vigilância Alimentar e Nutricional na atenção primária no município investigado (Uberlândia, Minas Gerais). Embora a maioria dos profissionais e dos coordenadores declare conhecer o sistema, seu uso como ferramenta de diagnóstico populacional e de apoio à tomada de decisão nas unidades de saúde permanece limitado. As práticas de manejo do aleitamento materno, ainda que presentes, mostram-se pouco sistematizadas, concentrando-se majoritariamente na primeira consulta do recém-nascido, com ênfase na orientação sobre a pega e a posição do bebê. Esses achados reforçam a necessidade de fortalecer ações voltadas à educação permanente, à ampliação da adesão à Estratégia Amamenta e Alimenta Brasil e ao aprimoramento do uso do Sistema de Vigilância Alimentar e Nutricional. Recomenda-se ainda que futuras pesquisas investiguem estratégias inovadoras de intervenção interprofissional, para o fortalecimento da prática clínica em aleitamento materno no contexto da atenção primária em diferentes regiões do país.
